# 3D bioprinting optimization of human mesenchymal stromal cell laden gelatin-alginate-collagen bioink

**DOI:** 10.1088/1748-605X/aca3e7

**Published:** 2022-12-08

**Authors:** Stephen W Sawyer, Kazuyo Takeda, Alaadin Alayoubi, Eman Mirdamadi, Ahmed Zidan, Steven R Bauer, Heba Degheidy

**Affiliations:** 1 Center for Biologics Evaluation and Research (CBER), U.S. Food and Drug Administration, Silver Spring, MD, United States of America; 2 Center for Drug Evaluation and Research (CDER), U.S. Food and Drug Administration, Silver Spring, MD, United States of America; 3 Department of Bioengineering, University of Maryland, College Park, MD, United States of America

**Keywords:** human mesenchymal stromal cells, MSCs, bioprinting, 3D printing, bioink, cellular encapsulation, gelatin-alginate-collagen

## Abstract

3D bioprinting technology has gained increased attention in the regenerative medicine and tissue engineering communities over the past decade with their attempts to create functional living tissues and organs *denovo*. While tissues such as skin, bone, and cartilage have been successfully fabricated using 3D bioprinting, there are still many technical and process driven challenges that must be overcome before a complete tissue engineered solution is realized. Although there may never be a single adopted bioprinting process in the scientific community, adherence to optimized bioprinting protocols could reduce variability and improve precision with the goal of ensuring high quality printed constructs. Here, we report on the bioprinting of a gelatin-alginate-collagen bioink containing human mesenchymal stromal cells (hMSCs) which has been optimized to ensure printing consistency and reliability. The study consists of three phases: a pre-printing phase which focuses on bioink characterization; a printing phase which focuses on bioink extrudability/printability, construct stability, and printing accuracy; and a post-processing phase which focuses on the homogeneity and bioactivity of the encapsulated hMSC printed constructs. The results showed that eight identical constructs containing hMSCs could be reliably and accurately printed into stable cross-hatched structures with a single material preparation, and that batch-to-batch consistency was accurately maintained across all preparations. Analysis of the proliferation, morphology, and differentiation of encapsulated hMSCs within the printed constructs showed that cells were able to form large,interconnected colonies and were capable of robust adipogenic differentiation within 14 d of culturing.

## 1. Introduction

According to the United Network for Organ Sharing, individuals waiting for organ transplants vastly outnumber the amount of organ donations received on a yearly basis. This trend has increased dramatically over the past few decades, creating the need for alternative solutions [1]. To meet this growing demand for transplantable organs, the interdisciplinary field of tissue engineering has seen rapid growth as researchers investigate ways to artificially create and repair damaged tissues [2]. Most recently, the advent of 3D printing has significantly enhanced tissue engineered solutions and has given credibility to the idea that tissue engineering could eventually lead to the creation of functional and implantable tissue constructs capable of mediating tissue regeneration and repair [3–5].

Bottom-up fabrication approaches such as additive manufacturing have proven extremely useful in the creation of structurally supportive biomaterial scaffolds containing patient specific geometries and interconnected porous networks that are able to facilitate tissue growth both *in vitro* and *in vivo* [6–8]. Investigations involving such techniques have generally relied on the printing of biomaterial scaffolds and the incorporation of cells post-print [9–11]. These types of approaches, however, could incur challenges when ensuring cells are equally distributed throughout the entirety of the printed constructs. Alternatively, a growing subset of additive manufacturing in which cells and biomaterials are printed in tandem has recently gained focus and has rapidly expanded to meet the needs of the regenerative medicine and tissue engineering communities. Called bioprinting, this strategy has resulted in the printing of several tissue constructs including multilayered skin [12], vascular tissues [13], artificial bones [14, 15] and cartilaginous structures [16].

The cell-delivery mediums employed in the bioprinting process, known commonly as bioinks [17], are either printed alongside other biomaterials or by themselves, and allow for cells to be printed in precise densities at specific locations within the printed tissues [18–21]. While there are numerous types of bioinks available, those containing combinations of naturally derived proteins such as collagen and gelatin and polysaccharides like alginate have gained wide usage in the field and have commonly been employed as the cell-containing mediums. In addition to allowing for the *in situ*-controlled distribution of cells within the printed constructs, these types of materials also provide the cells an environment that mimics the natural extracellular matrix (ECM) found *in vivo* and do not require cross-linking methods such as harsh chemicals or UV exposure that could unintentionally impact the viability and metabolic activity of encapsulated cells [21–26].

The types of biomaterials used to create bioinks not only play important roles in the rheological properties of the printed material, but also determine how seeded and encapsulated cells will adhere and interact within the final constructs. While it is important to choose combinations of natural and synthetic polymers that will closely mimic the ECM cells experience *in vivo*, care must be taken to ensure proper functional outcomes. Polysaccharides such as alginate, which are derived from brown algae, form long chain anionic polymers that aid cellular biocompatibility, allow for tunable viscosities, and provide construct stability through ionic crosslinking [27]. Hyaluronic acid (HA), a negatively charged polysaccharide common to the ECM and found in connective tissues such as the synovial fluid in joints, is an unbranched polysaccharide chain comprised of repeating disaccharide units containing ionizable groups that aid in cell binding, water retention, and cellular signaling [28]. In one recent study it was shown that through the deacetylation and sulfation of HA, binding of CD44+ cells to HA could be selectively decreased [29]. In yet another study, sulfate modifications of HA were utilized to show selective P-selectin and CD44 binding [30]. Alternatively, while some biomaterials necessitate modifications for cellular attachment, other naturally derived biomaterials such as gelatin and collagen readily support cell adhesion through various inherent functional groups. Additionally, as both gelatin and collagen are capable of being thermally crosslinked, culture conditions and printing parameters can be easily changed to fine tune fiber sizes and rheological properties of printed constructs [1].

Among the cell types that have been bioprinted for regenerative medicine and tissue engineering applications, human mesenchymal stromal cells (hMSCs) have gained significant attention due to their anti-inflammatory and immunomodulatory properties, their mesenchymal differentiation capacity, and their potential for self-renewal [31, 32]. Because of these characteristics, numerous studies have investigated whether MSCs support hematopoietic reconstruction and aid in the restoration and regeneration of tissues such as bone, cartilage, and muscle [33, 34]. While these properties alone are desirable for regenerative medicine and tissue engineering applications, the differentiation potentials of MSCs have also been shown to be improved when grown in a 3D environment [35–38], making them appealing candidates for bioprinting and subsequent 3D culture.

When using bioprinting technologies to fabricate cell-based constructs, numerous factors need to be considered to create a final product capable of being printed consistently and reliably. Depending on the desired final application of the printed constructs, every aspect of the printing process needs to be accounted for and optimized, from the basic printing parameters to the biological materials used. Factors such as rheological properties of the bioink, printing accuracy, printed construct stability, crosslinking strategies, biocompatibility, cell type, and encapsulated cell viability, proliferation, and metabolic activity all need to be assessed, in addition to the assays being used to measure the experimental outcomes [39–41]. To this end, it is important for the bioprinting process to be divided into three distinct phases in which the properties of the bioinks and printed constructs are evaluated and refined. These phases should include a pre-bioprinting phase which focuses on bioink selection, characterization, and printability; a printing phase which focuses on bioink extrudability, construct stability, and printing accuracy; and a post-processing phase which assesses the construct homogeneity and cellular bioactivity.

In this manuscript, characterizations performed at all three phases of the bioprinting process were used to create an optimized hMSC-laden, gelatin-alginate-collagen bioink capable of being 3D printed in a consistent and reliable manner. The preparation of the gelatin-alginate-collagen blend was standardized to minimize batch-to-batch variations, and rheological experiments were used to assist in the optimization of printing parameters to ensure printability and construct consistency. ImageJ analysis was used to analyze the accuracy of the printed constructs and an 3-(4,5-dimethylthiazol-2-yl)-5-(3-carbox ymethoxyphenyl)-2-(4-sulfophenyl)-2H-tetrazolium (MTS) assay was used to further assess print consistency as well as the homogeneity of the cellular distribution between constructs. Finally, post printing analysis via MTS and fluorescent microscopy was used to evaluate the ability of the encapsulated hMSCs to proliferate and differentiate after two weeks of culture.

## 2. Materials and methods

### 2.1. Preparation of gelatin-alginate-collagen bioink

To prepare the gelatin-alginate-collagen bioink (10% w/v gelatin/1% w/v alginate/0.8 mg ml^−1^ collagen), 0.5 g of gelatin powder (BioBots) and 0.05 g of sodium alginate (Alginic acid sodium salt from brown algae; Sigma-Aldrich; Cat#A1112) were dissolved separately in 1.5 ml of phosphate-buffered saline (PBS; Gibco; Cat#10010023) and heated to 70 °C for 30 min. After resting at room temperature for 15 min, the heating process was repeated two additional times. After the second heating, the gelatin and alginate solutions were mixed and left to rest for an additional 15 min prior to being heated a final time for 30 min. Next, 0.5 ml of prewarmed 37 °C 10× media (Gibco; Cat#11430030) was added to the gelatin-alginate solution and vortexed thoroughly. Upon vortexing, the gelatin-alginate solution was added to a 1.0 ml mixture (50:50 v/v) of neutralized rat-tail type I collagen (Corning; Cat#354249) and 4-(2-hydroxyethyl)-1-piperazineethanesulfonic acid (HEPES; pH 7.8; Sigma-Aldrich; Cat#90909C). The collagen-HEPES mixture was made directly prior to the addition of the gelatin-alginate solution by adding 0.5 ml of 4 °C collagen to 0.5 ml of room temperature HEPES buffer which had been previously adjusted to the proper pH through the dropwise addition of sodium hydroxide (NaOH). After mixing the gelatin-alginate-collagen solution, 0.5 ml of room temperature PBS without cells, or 0.5 ml of room temperature PBS with 5 × 10^6^ cells was added to the material and vortexed lightly before being transferred to a 30 cc syringe barrel (Nordson; Cat#7012134).

### 2.2. Endotoxin assay

Endotoxin concentrations within the gelatin-alginate-collagen solutions were assessed using a ToxinSensor^™^ Chromogenic LAL Endotoxin Assay Kit (GenScript; Cat#L00350 and Cat#L00350Y). Three independent acellular gelatin-alginate-collagen preparations were made as previously described and subsequently treated per the manufacturer protocols before being placed in 200 μl aliquots on a flat bottom 96 well tissue culture test plate (Millipore Sigma; Cat#Z707902). Aliquots of the independent preparations were read at *λ* = 545 nm on a multi-mode microplate reader (SpectraMax M Series Multi-mode Microplate Reader; Molecular Devices) and subsequently compared to the standard curve resulting from four kit standards (1, 0.5, 0.25, and 0.125 EU ml^−1^ respectively).

### 2.3. Cell culture

hMSC line PCBM1662 (All Cells, Emeryville, CA, www.allcells.com), was previously expanded and frozen as described [42]. A vial (1 million cells in 1 ml cryopreservation media) at passage 3 was thawed and gently added to 5 ml of prewarmed Minimum Essential Medium *α* (MEM *α*; Gibco; Cat#12561072) supplemented with 1% L-Glutamine (200 mM; Gibco; Cat#25030081), 1% penicillin streptomycin (Gibco; Cat#15140122), and 20% Fetal Bovine Serum (FBS; JM Bioscience; Lot1032). The cell solution was gently mixed and seeded equally within six T-175 Flasks (NUNC^™^; Cat#178883) containing 20 ml of prewarmed complete media. The flasks were maintained at 37 °C in a humidified 5% CO_2_ atmosphere (Sanyo MCO-18AIC CO_2_ Incubator; SANYO Electric Co., Ltd, Japan). Media was refreshed every 3 d until the flask reached 90% confluency, upon which the cells were detached from the flasks using 0.05% trypsin-EDTA (Gibco; Cat#25300054), counted, and resuspended in complete media to a final concentration of 10 × 10^6^ cells ml^−1^. This cellular concentration was subsequently diluted when added to the gelatin-alginate-collagen mixture for a final concentration of 1 × 10^6^ cells ml^−1^.

### 2.4. 3D printing and post-print cell culture

Gelatin-alginate-collagen bioinks both with and without cells were transferred into a sterile, 3 cc syringe barrel (Nordson; Cat#7012134) and placed in a 20 °C printhead on a 3D-Bioplotter (EnvisionTEC, Dearborn, MI) immediately after preparation and left to equilibrate to the desired printing temperature for 30 min. After reaching 20 °C, the material was printed using a 22 G needle (Nordson; Cat#7018298) into sterile 50 mm glass bottom culture dishes (1.5 coverslip; 30 mm glass diameter; MatTek; Cat#P50G-1.5–30-F). Once printed, the culture dishes containing the printed constructs were transferred to a BSL-2 hood and physically crosslinked with sterile, prefiltered 3% calcium chloride (CaCl_2_; Sigma-Aldrich; Cat#C1016) solution for 3 min. After crosslinking, the CaCl_2_ solution was gently aspirated, the constructs were rinsed three times with 1xPBS, and complete cell culture media was added. The constructs were maintained at 37 °C in a humidified 5% CO_2_ atmosphere. Half of the spent media was replaced with fresh media the first day after printing and half media changes were repeated every other day for the duration of the experiments using either complete media or adipocyte differentiation medium (Zenbio; Cat#DM-2). If the differentiation medium was used, it was introduced during the first half media change the day after printing.

### 2.5. Rheology

The mechanical and viscoelastic properties of the bioink and printed constructs were assessed using a Discovery HR-3 Hybrid Rheometer (TA Instruments Ltd, New Castle, DE). A 25 mm Peltier aluminum parallel plate containing a gap size of 1500 μm was used and the rheometer temperature control system was set to 20 °C. The bioink was initially assessed using a flow temperature ramp to determine the gelling point. The temperature ramp test was conducted from 37 °C to 15 °C with a ramp rate of 1 °C min^−1^ and shear rate of 4 s^−1^. Additionally, an oscillation amplitude sweep test was performed on the bioink and constructs before and after crosslinking to determine the linear viscoelastic region. The elastic modulus (G′) and viscous modulus (G″) in the linear region was used to characterize the structure of the bioink prior to deformation and was performed before running the oscillation frequency test needed to determine the shear rate. An oscillation amplitude test was carried out at 20 °C with a soak time of 60 s and strain range from 0.01% to 100%. The frequency was set at 1 Hz and five points per decade were collected. Once the shear rate point was determined from the linear viscoelastic region, the frequency test was performed in the range from 0.01 to 16 Hz.

All rheological measurements were carried out in triplicates. Data was analyzed using the TA Rheology TRIOS software (Version 3.0, TA Instruments Ltd, New Castle, DE).

### 2.6. ImageJ analysis

For each printed construct, a brightfield image was taken (14×; Leica Z6 APOA; Leica, Germany) for ImageJ National Institutes of Health (NIH) analysis. The image of the 3D printed grid (one per printed construct) was divided into 12 equal parts manually via ImageJ. Each partition contained two line features (L1, L2), two inner box diagonals (IN1, IN2), and a total length (T) that spanned the entire partition. All five features were subsequently measured using ImageJ and adjusted to a percent error based on the theoretical design measurements. Once adjusted, a 96 × 5 numeric matrix was generated for a set of eight images (eight constructs/images per experiment/print, 12 partitions per image, five features) for analysis. This process was repeated using three separate material preparations for three independent experiments/prints.

### 2.7. MTS cell proliferation assay

Metabolic activity directly after printing (baseline cellular density) and metabolic activity over time (cell proliferation) was assessed using a Celltiter 96 Aqueous Non-Radioactive Cell Proliferation Assay (MTS; Promega; Cat#G5421) following manufacturer protocols. The aqueous reagents were thawed and mixed prior to the assay being performed and diluted per the manufacturer protocol in complete media. Prior to the assay being performed, the culture media was carefully aspirated from the printed constructs, followed by two washes with PBS. 5 ml of MTS reagent and media mixture were added to the constructs, and the constructs were maintained at 37 °C in a humidified 5% CO_2_ atmosphere for 2 h. Throughout the duration of the incubation, the constructs were gently agitated at 60 RPM using a multi-platform shaker (Fisher Scientific Multi-Platform Mixer/Shaker-13687700; ThermoFisher Scientific) to ensure complete dissolution of the constructs. Upon completion of the incubation there was no residual debris from the constructs. Accordingly, six 130 μl aliquots of solution from each construct were taken, placed in a flat bottom 96 well tissue culture test plate (Millipore Sigma; Cat#Z707902) and read at *λ* = 490 nm on a multi-mode microplate reader (SpectraMax M Series Multi-mode Microplate Reader; Molecular Devices). Printed acellular constructs were used as controls for each experiment to normalize the data.

### 2.8. Fluorescent staining

#### 2.8.1. Cell viability

Cell viability of encapsulated MSCs was observed directly after printing using a live/dead assay. Constructs were stained with calcein-AM (live, 1:2000 dilution in 5 ml of complete media; Invitrogen; Cat#C1430) and ethidium homodimer (dead, 1:500 dilution in 5 ml of complete media; Invitrogen; Cat#E1169) directly after printing and maintained at 37 °C in a humidified 5% CO_2_ atmosphere for 1 h prior to being imaged via multiphoton microscopy (Leica SP8 multiphoton microscope; Leica Microsystems, Germany). Samples were counterstained and imaged with Hoechst 33342 (Hoechst; 2 μg ml^−1^; Invitrogen; Cat#H1399).

#### 2.8.2. Cell density and morphology

Cell density of encapsulated MSCs was evaluated directly after printing using calcein-AM. Constructs were stained with calcein-AM (1:2000 dilution in 5 ml of complete media; Invitrogen; Cat#C1430) directly after printing and maintained at 37 °C in a humidified 5% CO_2_ atmosphere for 1 h prior to being imaged via multiphoton microscopy. Likewise, cellular morphology of encapsulated MSCs was evaluated using the same procedure both one week and two weeks after printing.

#### 2.8.3. Adipogenesis

Constructs incubated with adipogenic media for two weeks were evaluated using a lipid stain (AdipoRed^™^; Lonza; Cat#PT-7009). Prior to imaging via multiphoton microscopy, the culture media was carefully aspirated from the printed constructs, and 5 ml of media containing 140 μl of stain per the manufacturer protocol was added. The constructs were maintained at 37 °C in a humidified 5% CO_2_ atmosphere for 1 h prior to imaging. Samples were counterstained with Hoechst (2 μg ml^−1^; Invitrogen; Cat#H1399).

### 2.9. Statistical analysis

Numerical data was entered into Microsoft Excel to calculate the mean and standard deviation and either two-sample *t*-test or one-way ANOVA was used to assess statistical significance of differences. *P*-values ⩽ than 0.05 were accepted as statistically significant. The coefficient of variation (CV) was also calculated where appropriate and expressed as percentages with values ⩽20% within experiments and 30% across experiments considered acceptable.

## 3. Results

### 3.1. Pre-bioprinting phase

#### 3.1.1. Bioink preparation

**Figure 1.**
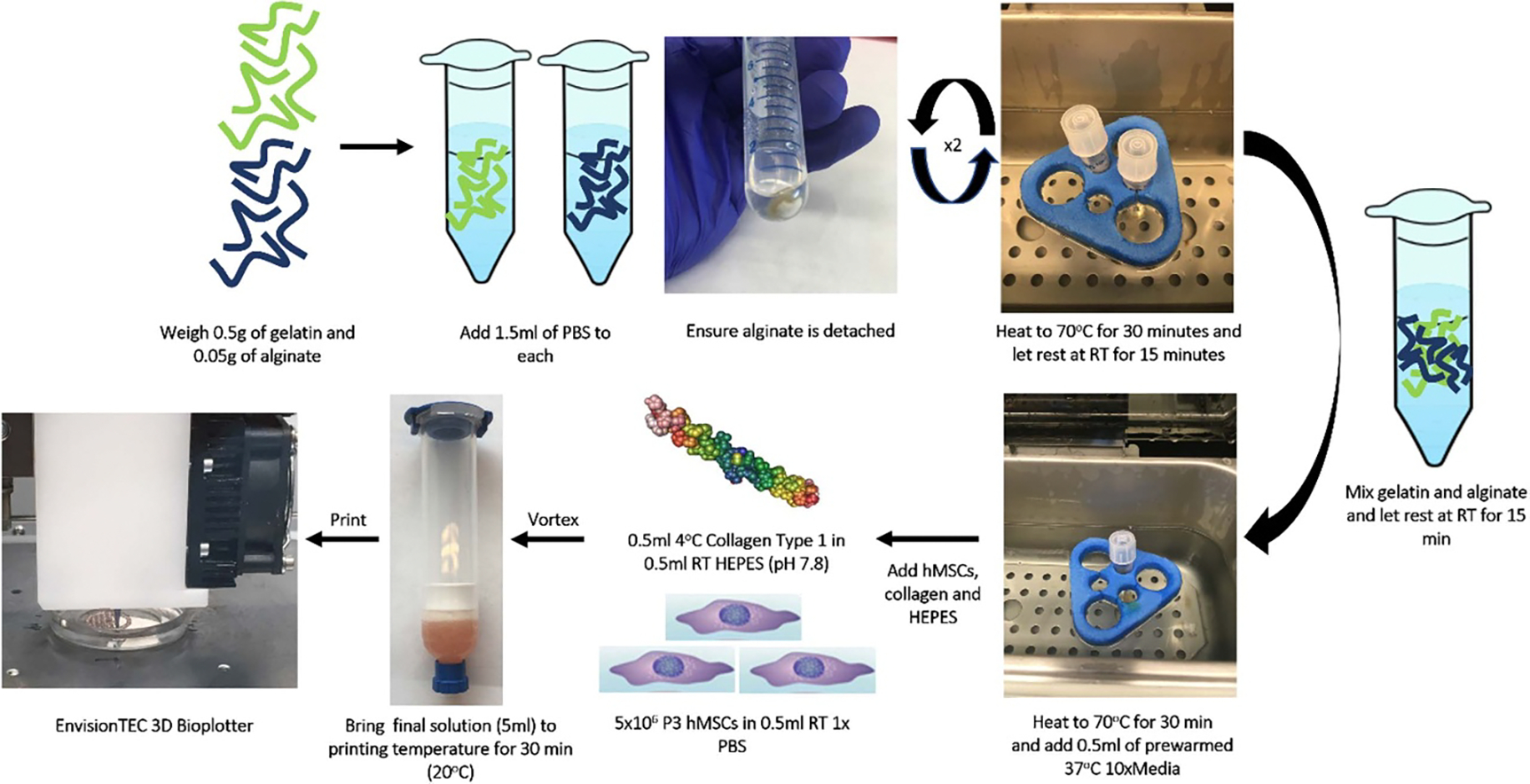
Material preparation. Representative diagram of the material preparation process as described in the materials and methods section. Gelatin and alginate powders were separately weighed and dissolved in PBS prior to being mixed aseptically in a biosafety cabinet. Once the gelatin-alginate solution was heated a third time, prewarmed 10× cell culture media, a mixture of collagen and HEPES buffer, and the cell suspension were added within the biosafety cabinet as described in the materials and methods prior to being transferred to the 3D Bioplotter (RT = room temperature).

In the process of developing the bioink, certain issues needed to be optimized to ensure proper bioink homogeneity as well as reduce variability that may arise from each material preparation (figure 1). It was important that each gelatin-alginate-collagen preparation, both with and without cells, was made on the day of printing to reduce variability that could occur during either short- or long-term storage. During the manufacturing process, the initial addition of phosphate-buffered saline to the gelatin powder and sodium alginate powder did not completely dissolve the material, so the preparation was subsequently heated to 70 °C and cooled three times to completely mix the materials and reduce the bioburden of the gel. After the second iteration of heating and cooling, the alginate and gelatin were capable of being added together to form a homogenous mixture. Due to the alginate solution being non-viscous and easily transferable, alginate was added to the gelatin prior to the iteration.

As the gelatin-alginate mixture cooled, the material viscosity increased, making it important to standardize the procedure and subsequent timing of the addition of the neutralized collagen and cell mixture (or PBS containing no cells). Once the third heating process of the gelatin-alginate solution was complete, the material was transferred to a 37 °C water bath while the cell culture area was prepared, thereby lowering the temperature of the material to one suitable for the addition of living cells while maintaining a less viscous state needed for homogenization. Once the cell culture area was prepared, prewarmed 37 °C 10× media was added to the gelatin-alginate, directly followed by neutralized 4 °C collagen. After the addition of collagen, PBS either with or without cells was added and the entire solution was gently vortexed to ensure all components were evenly distributed within the syringe barrel, creating a homogenous mixture to be transferred to the 3D printer. Visual evaluation throughout the process did not reveal any sign of bacterial or fungal contamination and endotoxin assays on acellular material preparations revealed that only relatively low levels of endotoxin were present (*n* = 3; 1.04 ± 0.01 EU ml^−1^).

#### 3.1.2. Material analysis

**Figure 2.**
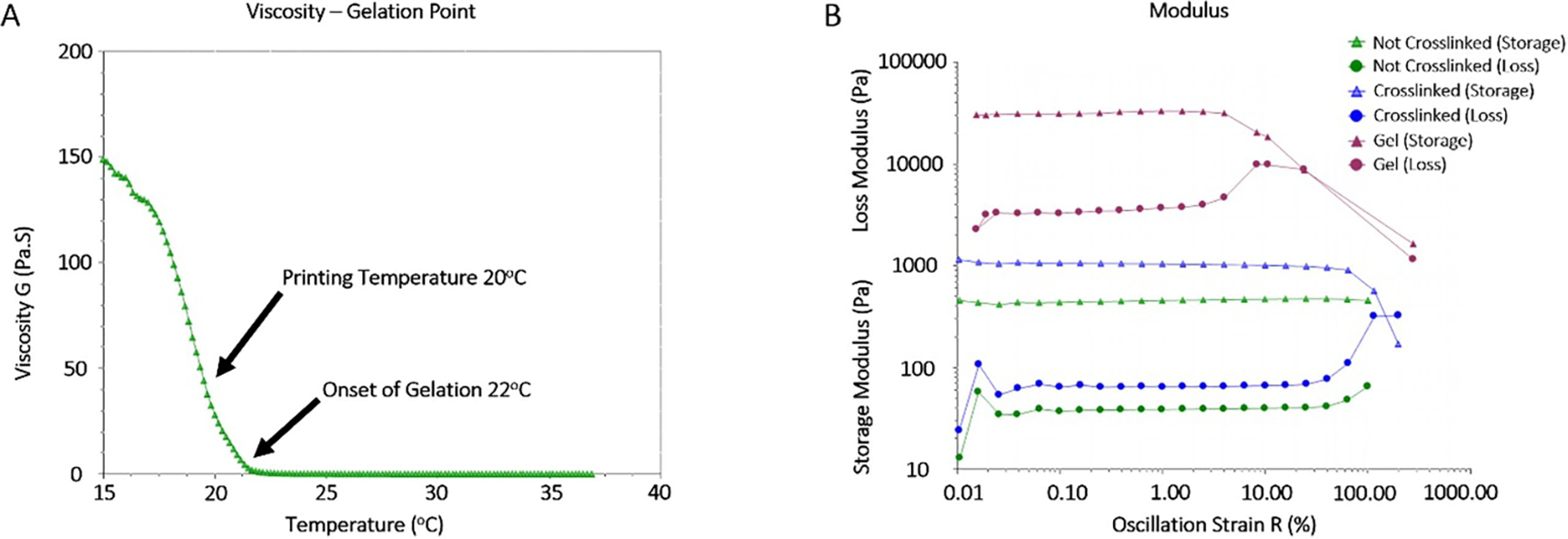
Material analysis. (A) A temperature ramp test of the material was performed from 37 °C to 15 °C at 1 °C intervals and the viscosity (G; Pa. S) was plotted as a function of temperature (^o^C) in order to determine the onset of gelation as well as the optimal printing temperature (*n* = 3). (B) The storage modulus (G′; Pa) and loss modulus (G″; Pa) of non-crosslinked printed constructs (green), crosslinked printed constructs (blue), and non-printed material (grey) were measured in response to a change in oscillation strain (%). The amplitude of the shear strain was varied between 0.01% and 100% while the frequency was maintained at 1 Hz to evaluate G′ and G″ of the samples (*n* = 3).

A temperature ramp test was performed to determine the sol-gel transition temperature of the cell-free bioink (figure 2(A)) and an initial viscosity of 17 mPa s was recorded at 37 °C. Afterwards, a slight increase in viscosity was recorded at lower temperatures, reaching 20 mPa s at 22 °C. The onset of gelation was recorded at 22 °C, where an increase of viscosity was observed as the temperature continued to be lowered. Afterwards, a gradual increase in viscosity was measured reaching a value of 150 Pa s at 15 °C. Based on these results, the oscillation tests were performed at 20 °C where the cell-free bioink was in the gelling phase but not too viscous to handle.

In the oscillation amplitude sweep test, the amplitude of the shear strain was varied between 0.01% and 100%, while the frequency was kept constant at 1 Hz to evaluate the storage (G′) and loss (G″) moduli of the samples (figure 2(B)). The plateau value of G′ in the linear-viscoelastic (LVE)-region represented the rigidity of the samples at rest while the plateau value of G″ represented a measure of the viscosity of the unsheared samples. At the initial oscillation strains, G′ and G″ showed constant values in LVE-region, indicating that the sample structures were undisturbed at these levels of mechanical strain. As soon as the moduli began to decrease, the structures became disturbed, and the end of the LVE-region was reached. Both crosslinked and un-crosslinked samples were shown to have similar and longer viscoelastic regions than those of the original printing material, clearly demonstrating their higher rigidities.

### 3.2. Printing phase

#### 3.2.1. Print consistency and imageJ analysis

Based on the results from the cell-free gelatin-alginate-collagen bioink characterization, it was determined that a printing temperature of 20 °C was optimal as the material was in the gelling phase but not overly viscous. However, although the material was printable, the printing pressure and speed needed to be optimized to reduce variability in the final printed constructs.

The first step in optimizing the printing speed and pressure was determining the minimum extrusion pressure for the cell-free bioink after cooling to 20 °C. After letting the material incubate at 20 °C for 30 min, it was found that a pressure of 1.2 bar was the lowest possible pressure capable of extrusion at any given print speed. However, while cell-free bioink was capable of being printed, this pressure resulted in highly inconsistent lines and clumping.

Beginning at a speed of 7 mm s^−1^ the pressure was gradually increased to determine which pressures produced consistent lines with minimal clumping. Pressures of 1.7–1.9 bar produced consistent lines and allowed for the creation of cross-hatched constructs consisting of five layers. However, as the printing pressure continued to be increased, the amount of material extruded was in excess and led to areas of the print being undesirably filled between the hatches. Accordingly, increasing the speed of the print to either 8 or 9 mm s^−1^ reduced the amount of filling at the higher pressures and constructs printed at 1.8 bar at both these speeds led to printed structures that were reproducible with no excess filling or clumping.

To determine whether a print speed of 8 or 9 mm s^−1^ would be used going forward, samples were printed at both speeds under a pressure of 1.8 bar, physically crosslinked in a CaCl_2_ solution, and stored in complete culture media for two weeks, as described in the methods. After 2 weeks of culture, constructs printed at 9 mm s^−1^ and 1.8 bar were far less stable than those printed at 8 mm s^−1^ and 1.8 bar. Unlike the 8 mm s^−1^ printed constructs, numerous trapped air bubbles were observed between the hatches of the 9 mm s^−1^ constructs that were attributed to the slightly thinner lines, indicating there may have been incomplete CaCl_2_ crosslinking after the initial printing. Accordingly, a print speed of 8 mm s^−1^ at a pressure of 1.8 bar was determined to be optimal for printing the gelatin-alginate-collagen material at 20 °C. Additionally, visual inspection of the printed constructs after 14 d of culture consistently indicated no microbial contamination.

**Figure 3.**
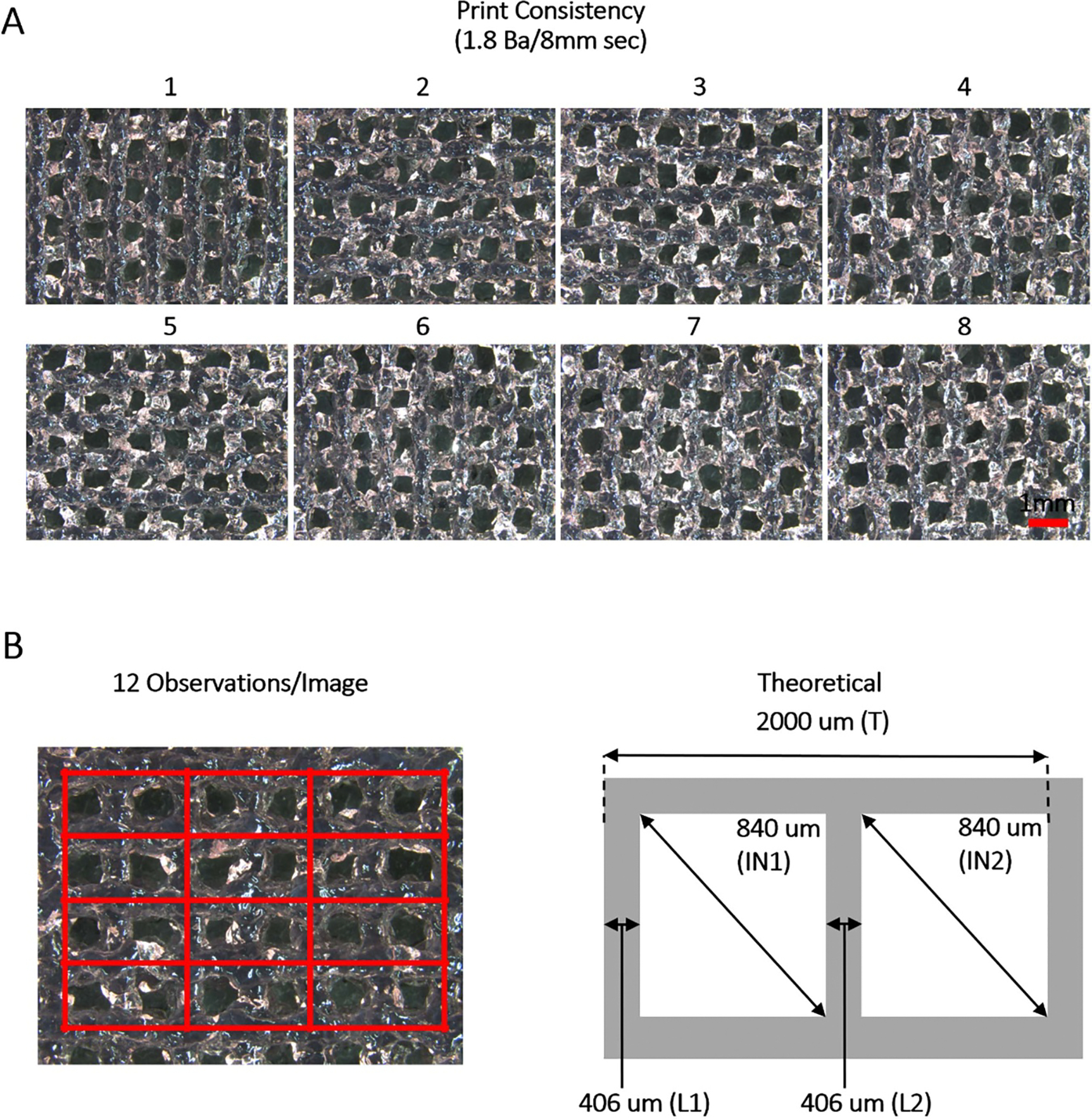
Print consistency. (A) Printing 5 ml of material at 1.8 bar at a speed of 8 mm s^−1^ allowed for the printing of eight total constructs and utilized the entirety of the prepared stock (brightfield images taken at 14× in the center of each construct; scale bar = 1 mm). (B) Printed construct images were broken into 12 measurable sections (red boxes; left of panel (B)). Within each individual section, five features were identified to be measured via ImageJ and compared to their theoretical values (*L*1, *L*2, IN1, IN2, *T*; right of panel (B); measurements shown as μm). Three separate experiments (prints) containing eight constructs per print were analyzed with final averages and standard deviations for each feature summarized in table 1 in the text. CV values represent total variance for each feature across all prints. (*n* = 3; 96 observations per feature per print; CV ⩽ 30% considered significant).

At the desired temperature, speed, and pressure, eight constructs could reproducibly be printed with 5 ml of cell-free bioink (figure 3(A)). To determine the consistency of the prints, three independent cell-free material preparations and prints were conducted, and images of the constructs were subjected to image analysis via ImageJ (figure 3(B)). After segmenting the images into 12 sections (red boxes), the five printed features (IN1, IN2, L1, L2 and T) were maintained overall when compared to the theoretical design measurements (table 1). While the inner diagonals of the printed structure deviated approximately 20% from their theoretical counterparts, the printed lines themselves varied by approximately 10%, indicating that the prints from different material preparations were similar, consistent, and accurate.

### 3.3. Post-processing phase

#### 3.3.1. Baseline cellular density and construct homogeneity

**Figure 4.**
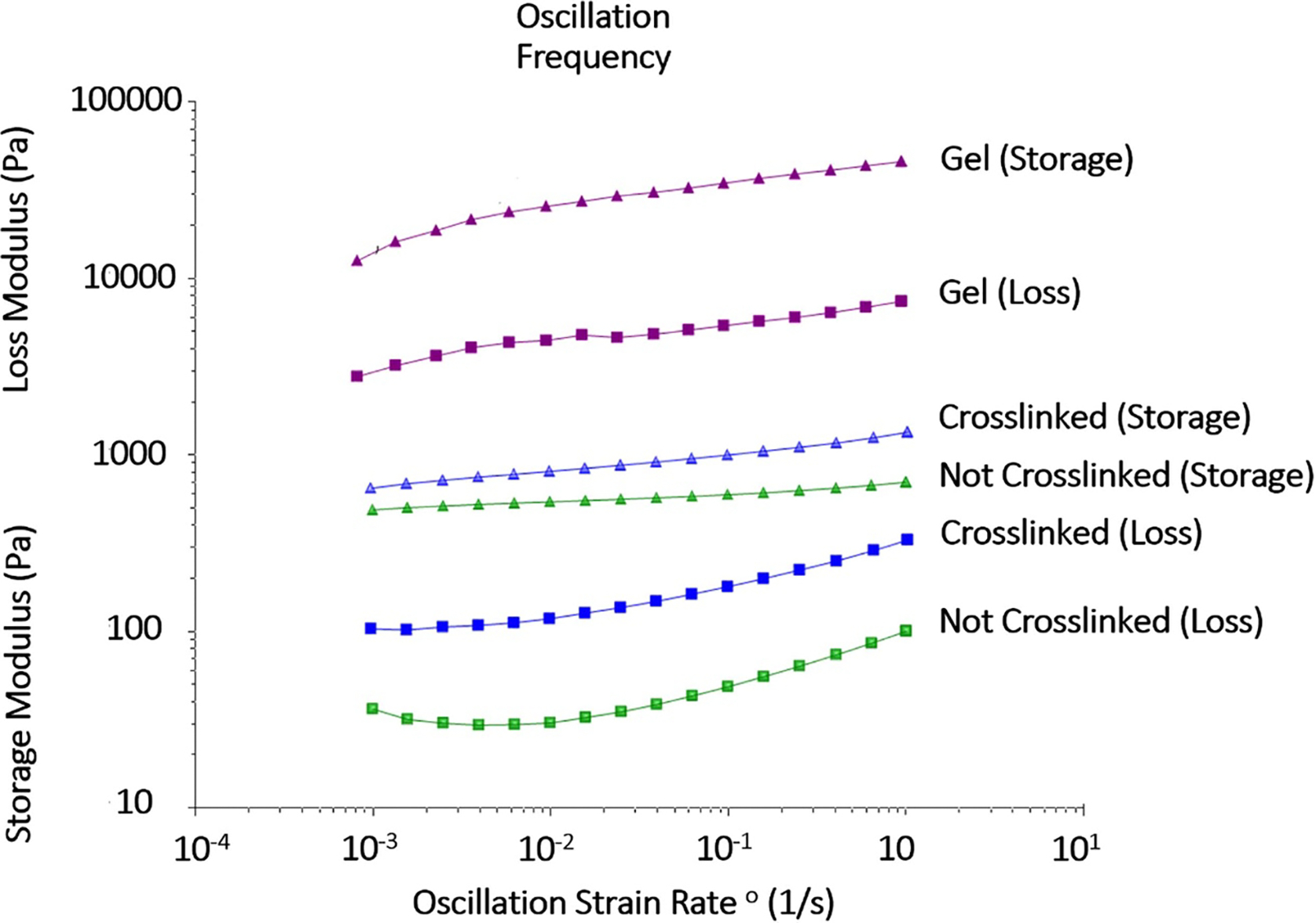
Cell sedimentation. An oscillation frequency sweep was performed on the material and the storage modulus (G′; Pa) and loss modulus (G″; Pa) of non-crosslinked printed constructs (green), crosslinked printed constructs (blue), and non-printed bioink (grey) were plotted as a function of the oscillation strain rate (1 s^−1^) to determine the sedimentation stability for potential material dispersions (*n* = 3).

After confirming the reproducibility of the printed constructs, an oscillation frequency sweep test was performed on the cell-free bioink to describe the sedimentation stability of dispersions within the gel, such as cells (figure 4). Using a constant amplitude value of 1% that was chosen from the LVE region of the previously discussed amplitude test, the frequency was screened between 0.01 and 16 Hz. At the lower frequencies, all measured G′ values were observed to be higher than their G″ counterparts, indicating that dispersed particles would remain suspended in the material during handling and storage and therefore be unlikely to sediment.

**Figure 5.**
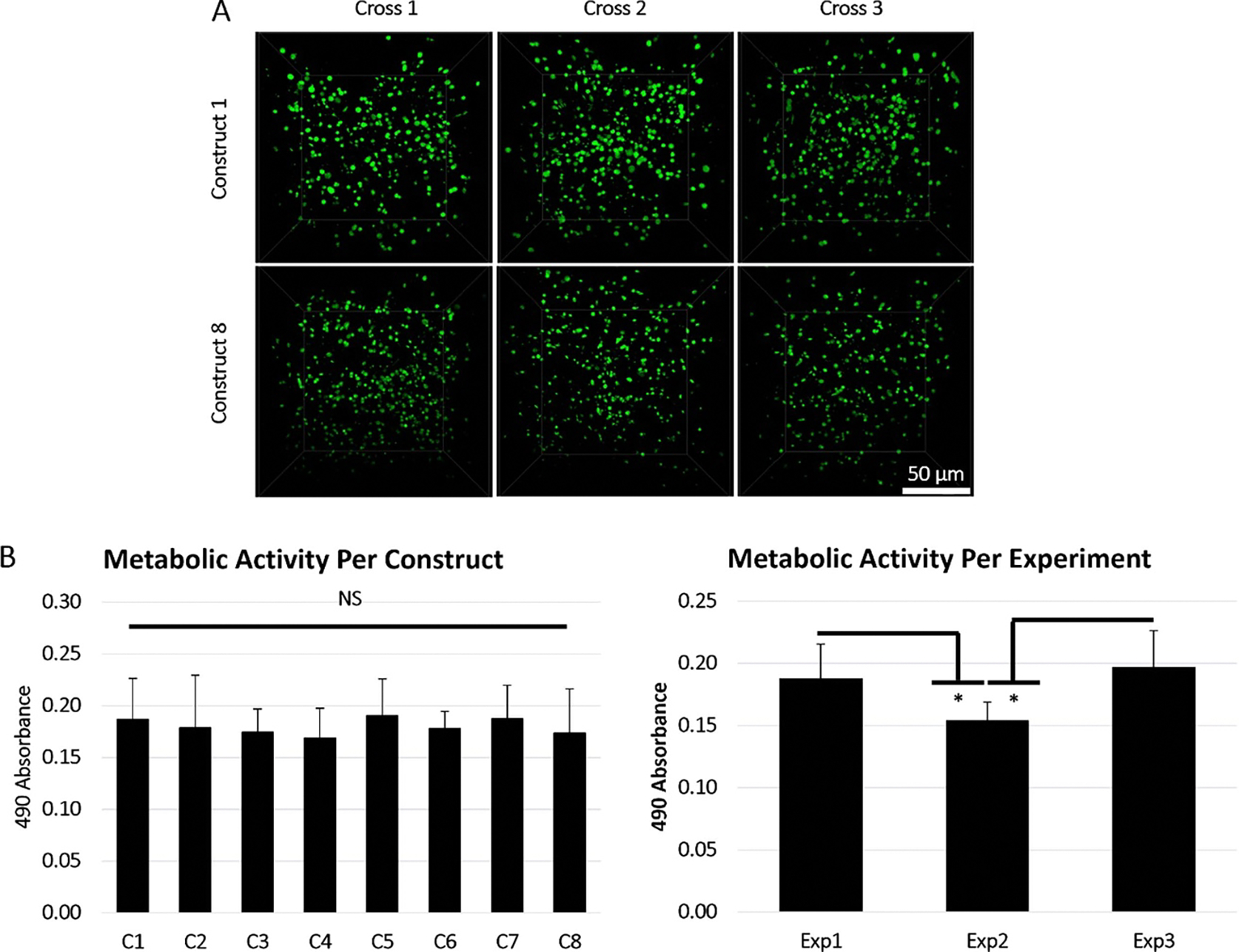
Cellular distribution and metabolic activity post printing. (A) Cells encapsulated within printed gelatin-alginate-collagen constructs were stained with Calcein (green) post printing to determine the uniformity of cell distribution between constructs. Images were taken via fluorescent multiphoton microscopy from the first and last printed constructs (images taken randomly in crossed sections of construct; scale bar = 50 μm). (B) Colorimetric MTS cell proliferation assay administered directly after printing to assess the uniformity of metabolic activity between printed constructs and experimental prints. Final averages, standard deviations, and CV values are summarized in table 2 in the text. (*n* = 3; * = *p*-value ⩽ 0.05; one-way ANOVA performed on B left; two-sample *t*-tests performed on B right).

Passage 3 hMSCs were added to the bioink preparations as described in the material and methods and constructs were printed at 20 °C under 1.8 bar with a speed of 8 mm s^−1^. To determine if the distribution of cells would be consistent between each of the eight printed constructs, the encapsulated hMSCs were stained with calcein-AM and imaged via multiphoton microscopy immediately after printing to observe viable cell dispersion (figure 5(A)). Viable cells appeared to be equally distributed between the constructs as predicted by the oscillation sweep test.

To quantitatively confirm that the total number of cells from construct to construct was reproducible and that cell settling did not occur during the print duration, an MTS assay was used. As the MTS assay relies on the production of formazan product based on the reduction capacities of live cells, it effectively considers all viable cells within the constructs and prevents errors that could be associated with counting cells manually via randomized images of a three-dimensional space. Accordingly, three independent bioink preparations and experimental prints were conducted and the reduction capacities of the live cells were measured immediately after printing and crosslinking. The resulting data was analyzed two different ways: examining the metabolic activity from construct 1 to construct 8 averaged across each experiment (figure 5(B) Left) and examining the total metabolic activity within the three independent experimental runs (figure 5(B) Right). An acceptable amount of variation in the total metabolic activity was observed both within and across the experimental runs (CV ⩽ 20% within experiments; CV ⩽ 30% across experiments) (table 2), thereby confirming the observations from the imaging data and rheological experiments. To further corroborate the initial observations, a one-way ANOVA was used to confirm that the metabolic activity of constructs 1 through 8 averaged across each experiment were statistically similar. However, two-sample *t*-tests used to assess the statistical differences within the three experiments showed that while experiments 1 and 3 were statistically identical, the total metabolic activity in the second experiment did deviate (*p* > 0.5) from the other two, indicating that the initial encapsulation process could impact the metabolic activity directly after printing, regardless of how statistically similar the individual print runs were.

#### 3.3.2. Encapsulated cellular bioactivity within printed constructs

**Figure 6.**
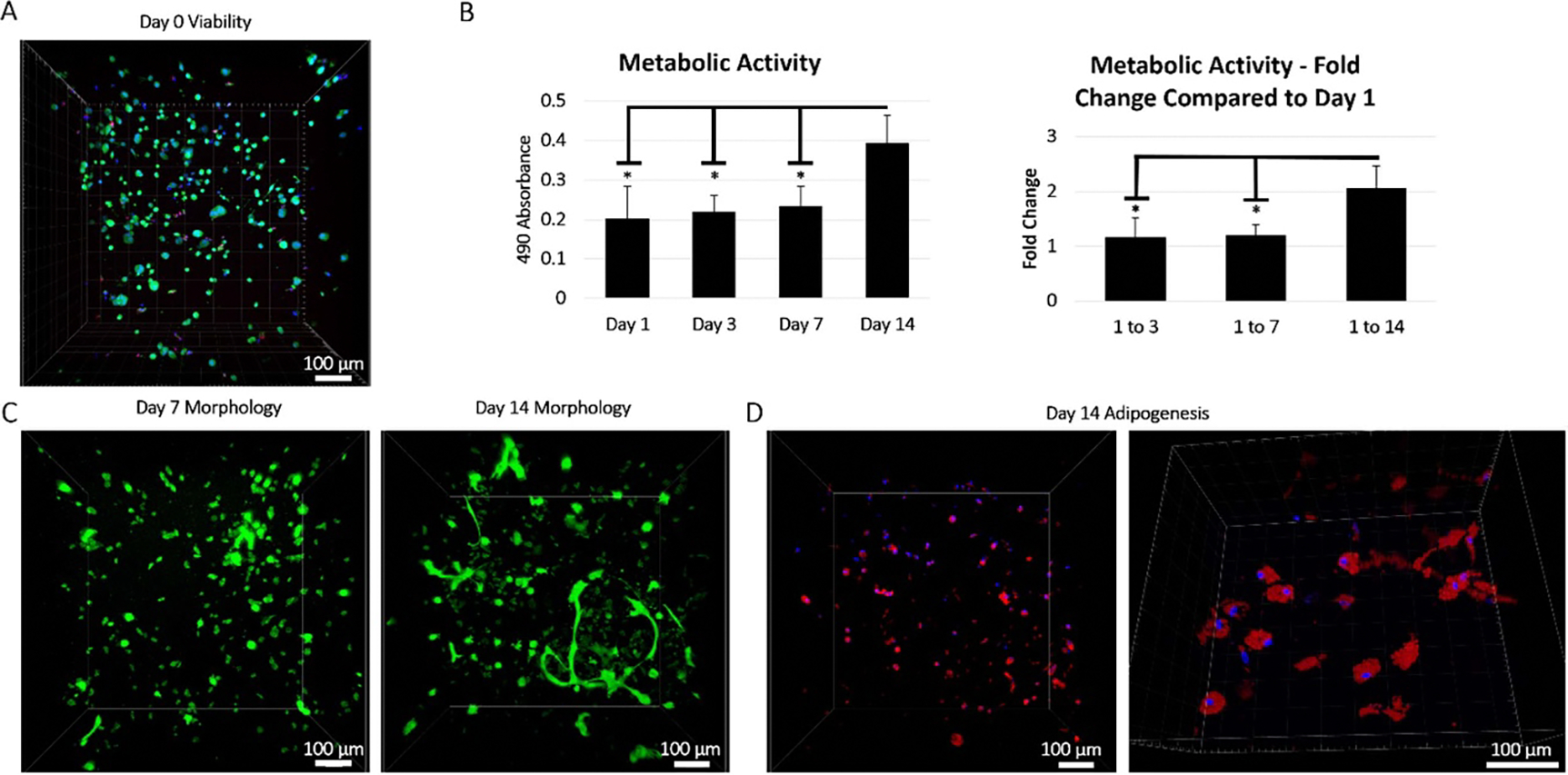
Cellular activity. (A) Cells encapsulated within printed gelatin-alginate-collagen constructs showed high viability (green) and minimal cell death (red) immediately after printing. Cell nuclei were counterstained with Hoechst (blue) (scale bar = 100 μm). (B) Colorimetric MTS cell proliferation assay was performed on constructs after 1, 3, 7, and 14 d of culture post print and the 490 absorbance was plotted as a function of time to assess the metabolic activity of the encapsulated cells over the two-week period (left; *n* = 4; ∗ = *p*-value ⩽ 0.05 via two-sample *t*-test). Fold change in the metabolic activity of encapsulated cells within constructs at days 3, 7, and 14 as compared to day one after printing was plotted to assess when cells began to significantly proliferate over the two week period (right; *n* = 4; * = *p*-value ⩽ 0.05 via two-sample *t*-test). (C) Cells within the printed constructs were stained with Calcein (green) and imaged via fluorescent multiphoton microscopy after 7 (left) and 14 (right) days of culture to assess the morphological features of the encapsulated cells (scale bar = 100 μm). **(D)** 1 d post printing, encapsulated cells were induced to differentiate into adipocytes and showed robust lipid formation (red) after 14 d of culture via fluorescent Nile red staining. Cells were counterstained with Hoechst (blue) (scale bar left = 100 μm; scale bar right = 100 μm).

Directly after printing and crosslinking, cells were viable as established via the MTS assay and live/dead staining confirmed that more than 80% of the hMSCs were living (figure 6(A)). To determine whether there were long term effects caused by the printing process on the encapsulated hMSCs, the MTS assay was again used to determine if the cells remained viable and metabolically active over the course of two weeks (figure 6(B)). During the first week of culture, metabolic activity did not increase or decrease significantly, indicating that the viable cells were not apoptotic and still capable of proliferation. After the first week of culture, however, the measured metabolic activity began to increase rapidly and showed a two-fold increase in activity at 14 d. These results were reinforced via fluorescent confocal microscopy after both 1 and 2 weeks of culture with cells appearing to still be spherical in shape at 7 d but showing characteristic spreading and colony formation at 14 d (figure 6(C)). Additionally, cells induced to undergo adipogenesis showed little to no differentiation after 7 d of culture but robust lipid formation two weeks after printing (figure 6(D)).

## 4. Discussion

In this investigation, the bioprinting process was divided into three distinct phases in which the properties of the bioink and printed constructs could be evaluated and refined to reduce variability from print to print. As numerous studies have shown that mechanical interaction with the microenvironment and cell adhesion are two of the most important factors affecting MSC differentiation [43–48], the types of polymers and their respective ratios were critical factors in bioink preparation. While combinations of gelatin and alginate have been commonly used in bioprinting processes, such systems have proven to be unreliable in terms of bioactivity due to cells being unable to properly degrade the gelatin/alginate matrix [49], necessitating the need of a third polymer that would support encapsulated cells. Appropriately, collagen proved to be essential in the preparation of our bioink as it supported the biological activity of the encapsulated hMSCs. To this end, in the pre-printing phase we derived the preparation of a gelatin-alginate-collagen bioink, the components of which have been well documented in the literature as prominent bioink candidates [27, 50, 51]. For example, a recent study showed that a tripolymer combination of these biomaterials allowed for the bioprinting of human corneal epithelial cells within constructs that supported high cell viability and proliferation [52]. Collagen, which is the main component of the ECM in organs such as bone, muscle, skin and cartilage, is also the primary structural component to which cells engage in order to adhere and migrate [53] and was an important addition to our material.

The preservation of aseptic conditions in the presence of viable cells was another important consideration for the bioink preparation in the pre-printing phase. While heating to high temperatures, irradiation, and chemical treatments have all been used to sterilize biomaterial scaffolds and post-printed constructs [54], the incorporation of cells within the bioink itself introduces additional factors to consider. In such cases involving cell-laden bioinks, researchers can incorporate high temperatures, filtration steps, or irradiation prior to the addition of cells, but the integrity of the material would need to be confirmed. However, low temperature treatments have been shown to be suitable prior to cellular incorporation by briefly heating the material to 70 °C multiple times, thereby suitably denaturing bacterial strains but maintaining bioink printability [55]. Furthermore, in the case of the gelatin-alginate-collagen bioink used in this investigation, the three rounds of heating used in the material preparation led to no evidence of contamination based on repeated visual inspection and aided in the mixing of the material to achieve bioink homogeneity. Additionally, although no contamination was visibly present, it was informative to perform an endotoxin assay on the material to determine whether it would be suitable in the future for possible *in vivo* studies. While it has been shown that endotoxin concentrations within hydrogels can vary considerably if not treated appropriately [56], we found that our acellular preparations consistently had low levels of endotoxins measuring approximately 1.04 ± 0.01 EU ml^−1^, which was only marginally above the test kit’s stated upper limit of 1 EU ml^−1^. Since each printed construct contained 1.6 ml of material, the total endotoxin concentration for each printed structure would be approximately 1.67 EU ml^−1^.

In many studies, it is common to choose a bioink and not report the optimization steps required to achieve suitable printability. While lack of information on optimization is not always the case, the material preparation and optimization should be adequately reported to ensure reproducibility and printability regardless of the bioprinting system used. For optimal functionality, constructs printed in any system need to meet certain mechanical requirements such as stiffness and retraction to maintain tissue continuity and stability in both the printing and post-printing phases [57]. In this study, a series of rheological tests were performed to determine suitable printing conditions for the gelatin-alginate-collagen bioink that could be used as a starting template for the printing of this bioink on different bioprinters. After determining that the optimal printing temperature was 20 °C, an oscillation amplitude sweep test was performed to determine the storage and loss moduli of the material and characterize the gel for other potential applications. The recorded storage moduli were larger than the loss moduli in samples tested which indicated that the samples behaved more like viscoelastic solids rather than viscoelastic fluids. Additionally, the crosslinked constructs measured higher G′ and G″ than non-crosslinked, which was expected as tighter network structures form at higher cross-linking densities [58]. All together, these results confirmed that the gelatin-alginate-collagen bioink printed at 20 °C in a cross-hatched fashion was reproducibly printable, would produce accurate constructs in our system, and could potentially provide a framework for others should they choose to use similar materials in different systems.

While the printability of the gelatin-alginate-collagen bioink was determined via rheology, it was also necessary to determine whether the extrudability of the material was sufficient to create printed constructs that were reproducible and statistically indistinct. In this investigation, we chose a combination of an iterative process and ImageJ analysis to determine that a print pressure of 1.8 bar at a speed of 8 mm s^−1^ resulted in nearly identical printed constructs that were statistically indistinguishable from batch to batch. Additionally, while incomplete crosslinking and lack of construct stability are two common issues encountered when employing bioinks requiring the ionic crosslinking of polysaccharides such as alginate [59–61], the print conditions used in this investigation allowed for sufficient CaCl_2_ crosslinking post-printing and stability in culture for up to two weeks.

When printing constructs containing encapsulated cells, it is important to ensure that the size and shape of the printed structures allow for proper nutrient distribution. As the natural diffusion range for nutrients within 3D systems is approximately 300 μm, the printed constructs must either contain interconnected porous networks or be designed in a way that naturally mitigates cellular hypoxia and necrosis [62–64]. By not adhering to such critical design features, 3D constructs in static culture systems may result in hypoxic cores and limited areas of bioactive and viable cells [65–68]. In this work, the theoretical dimensions for each printed line in the cross-hatched structure were 406 μm, thereby ensuring that cells would always be between 200 and 300 μm from a nutrient source. Additionally, the cross-hatched pattern could be easy scaled both up and down with no negative effect on nutrient distribution.

In conjunction with data establishing the extrudability and printing accuracy of the bioink, assessing the printed construct’s homogeneity and encapsulated cellular bioactivity in the post-printing phase should be accomplished through various assays that consider the entire construct, not just representative portions. In many instances, researchers provide qualitative data such as viability images, histological section staining, and immunohistochemistry images to validate the cellular bioactivity within the printed constructs [41], but fail to quantify the bioactivity of the entire construct.

In this investigation, qualitative data indicated that cells were viable and metabolically active post-printing, as indicated by the live/dead images, morphological images, and Nile-red staining. It should be noted, however, that if quantitative data were to be drawn from the fluorescent images, secondary staining using near infrared dyes or other dyes that absorb past 600 nm would help reduce potential artifacts caused by background noise. Conversely, by employing an MTS assay we were able to provide data encompassing the whole of the printed constructs that allowed for conclusions to be drawn regarding the homogeneity and cellular bioactivity of the printed material. Rheology data indicated that cell sedimentation during printing would not occur and that the cells would remain suspended in solution, and this conclusion was supported quantitatively via MTS by measuring the total metabolic activity of encapsulated cells per construct per print. Furthermore, as the similarity between printed constructs was established from batch to batch, the additional conclusion regarding the even distribution of cells per construct further advanced the idea that the qualitative images were more representative of the entire printed construct and not just randomized portions. The uniform, viable cell distribution determination was additionally confirmed by the MTS data showing conclusively that in the second week of culture, the metabolic activity in all constructs for all experiments increased by a factor of two, indicating that the cells were actively replicating and spreading.

## 5. Conclusion

Our data showed that we were able to optimize and consistently print gelatin-alginate-collagen constructs containing viable human MSCs via an optimized bioprinting process that was broken up in to three distinct phases. By investigating the printability and extrudability of our gelatin-alginate-collagen bioink, and by further ensuring that the material could be printed accurately and consistently, we were able to print eight cell-laden constructs per experiment that were stable in culture for up to two weeks and capable of supporting robust cellular proliferation and differentiation. In addition, our study showed that investigative approaches such as ours could potentially be used to help ensure that bioprinted constructs are homogenous from batch to batch and that these approaches may be suitable for tissue engineering applications.

## Figures and Tables

**Table 1.** Average of individual features.

Measurement	Theoretical value (μm)	Average value (μm)	Std. dev. (μm)	CV (%)

L1	406.00	367.40	45.40	10
L2	406.00	363.31	47.39	11
IN1	840.00	655.52	105.76	22
IN2	840.00	673.20	100.22	20
T	2000.00	1846.50	112.86	8

**Table 2.** Metabolic activity per construct and per experiment.

Metabolic activity per experiment							

		Exp 1	Exp 2	Exp 3	Average	Std. Dev.	CV (%)^b^

Metabolic Activity	Construct 1	0.17	0.16	0.23	0.19	0.04	21
Per Construct	Construct 2	0.15	0.15	0.24	0.18	0.05	28
	Construct 3	0.15	0.18	0.19	0.17	0.02	13
	Construct 4	0.19	0.14	0.18	0.17	0.03	17
	Construct 5	0.22	0.15	0.20	0.19	0.04	19
	Construct 6	0.20	0.17	0.17	0.18	0.02	9
	Construct 7	0.20	0.15	0.21	0.19	0.03	17
	Construct 8	0.22	0.15	0.15	0.17	0.04	25
	Average	0.19	0.15	0.20		490 Absorbance	
	Std. Dev.	0.03	0.01	0.03			
	CV (%)^a^	15	9	15			

a within experiment CV.

b across experiment CV.

## Data Availability

All data that support the findings of this study are included within the article (and any supplementary files).
